# A Case of Hyperglycemia-Induced Epileptic Homonymous Hemianopsia

**DOI:** 10.7759/cureus.65102

**Published:** 2024-07-22

**Authors:** Wataru Shiraishi, Yukiko Inamori, Yusuke Nakazawa, Hirofumi Shii

**Affiliations:** 1 Department of Neurology, Kokura Memorial Hospital, Kitakyushu, JPN; 2 Department of Internal Medicine, Shiraishi Internal Medicine Clinic, Nogata, JPN; 3 Department of Neurorlogy, Kokura Memorial Hospital, Kitakyushu, JPN; 4 Department of Neurology, Kitakyushu Yahata Higashi Hospital, Kitakyushu, JPN

**Keywords:** nonketotic hyperglycemia, occipital lobe seizure, “diabetes mellitus”, gadolinium enhancement mri, #seizure, hyperglycemia.

## Abstract

Hyperglycemia sometimes initially presents with neurological manifestations, including seizures, visual hallucinations, choreoathetosis, hemiballismus, myoclonus, tremor, and consciousness disturbance. Epileptic seizures induced by hyperglycemia are reported to occur predominantly in the occipital lobe, and the epileptic form is mainly epilepsia partialis continua. Of the two patterns of hyperglycemia (ketotic or nonketotic), nonketotic hyperglycemia is more commonly associated with seizures because ketosis has an anticonvulsive effect, so hyperglycemia-induced seizures are generally seen in nonketotic patients.

Here, we report a 51-year-old Japanese male with intermittent homonymous hemianopsia who presented high hemoglobin A1c (19.1%). He had been drinking 3 L of the sugared soft beverage every day. After admission, he showed left-sided hemiconvulsion. Anti-seizure medications and insulin treatment were administered, and his seizure was aborted. The magnetic resonance imaging (MRI) showed a high-intensity lesion in the diffusion-weighted image and fluid-attenuated inversion recovery with gadolinium enhancement in the occipital lobe.

In hyperglycemic convulsions, MRI sometimes shows leptomeningeal or parenchymal gadolinium enhancement. In addition, most hyperglycemic seizures are associated with nonketotic hyperglycemia and show occipital-dominant imaging abnormalities. We report this case by reviewing the differential diagnosis.

## Introduction

Hyperglycemia sometimes presents with neurological symptoms, including seizures, visual hallucinations, choreoathetosis, hemiballismus, somatosensory symptoms, headaches, and coma [1]. Seizures associated with hypoglycemia are well recognized, and hyperglycemia-induced seizures are also described in the literature [2-4]. While seizures associated with hypoglycemia are mainly generalized, those associated with hyperglycemia are typically focal motor seizures, often manifesting as epilepsia partialis continua [3,5]. One of the famous forms of seizures associated with hyperglycemia is nonketotic hyperglycemia-induced seizures (NKHS) [6-8]. NKHS causes mainly occipital lobe seizures as well as focal seizures [4]. In magnetic resonance imaging (MRI), NKHS sometimes represents T2 hyperintensity lesions in the cortical area associated with T2 hypointensity in the subcortical regions [4,8]. NKHS also sometimes shows gadolinium leptomeningeal or parenchymal enhancement [9]. Here, we present a patient with severe ketotic hyperglycemia showing occipital seizures presenting occipital brain lesion gadolinium enhancement. The patient consumed a soft drink containing 300 g of sugar for three months every day. Through this case, we will review the hyperglycemia-induced seizures and other differential diagnoses that can present similar imaging features.

## Case presentation

A 51-year-old, right-handed Japanese male presented with a five-day history of episodic, intermittent left homonymous hemianopsia. The patient intermittently reported experiencing brightness in the left visual field of both eyes. Three months before admission, he developed prostatitis and was advised by his family doctor to drink a sufficient amount of water. Subsequently, he began consuming 3 L of a sugared soft drink daily, which contained about 300 g of sugar. One month before admission, he experienced polydipsia, polyuria, and a weight loss of 15 kg. On physical examination, his height was 166 cm, body weight was 66 kg, body mass index (BMI) was 24 kg/m², blood pressure was 140/90 mmHg, heart rate was 70 bpm with a sinus rhythm, and body temperature was 36.2 degrees Celsius. He had no thyromegaly, skin lesion, or edema of extremities. On neurological examination, his Glasgow Coma Scale was 15 (eye, 4; verbal, 5; motor, 6). He claimed left homonymous hemianopsia. His facial and limb movements were normal. In the sensory system, he showed mild vibration disturbance his lower extremities. His deep tendon reflex, including Achilles tendon reflex, was normal. His plantar reflex was a downward response. In the autonomic nervous system, he experienced erectile dysfunction. On laboratory examination, his fasting blood glucose level was 266 mg/dL (normal, 70-109 mg/dL), hemoglobin A1c level 19.1% (normal, 4.6%-6.2%), and lactalbumin was 54.1% (normal, 11%-16%), showing severe hyperglycemia. Other laboratory data showed negative or within normal limits, including white blood cell count, hemoglobin, electrolytes, liver enzyme, coagulation, tumor markers, autoantibodies, and kidney function. Total ketones were 61 µmol/L (normal, <130 µmol/L), and ketosis was not observed (Table 1).

**Table 1:** Laboratory investigations. Blood tests showed a nonketotic hyperglycemic status.

	Results	Reference data
Hemoglobin (g/dL)	16.4	13.0-17.9
White blood cell (/μL)	6,200	3,000-8,900
Platelet (^10^4^/μL)	39.4	12.0-39.0
HbA1c (%)	19.1	4.6-6.2
Blood glucose (mg/dL)	279	70-109
Lactalbumin (%)	54.1	11.0-16.0
Total ketones (μmol/L)	61	<130
Acetoacetic acid (μmol/L)	41	<55
3-Hydroxybutyric acid (μmol/L)	20	<85

**Figure 1:**
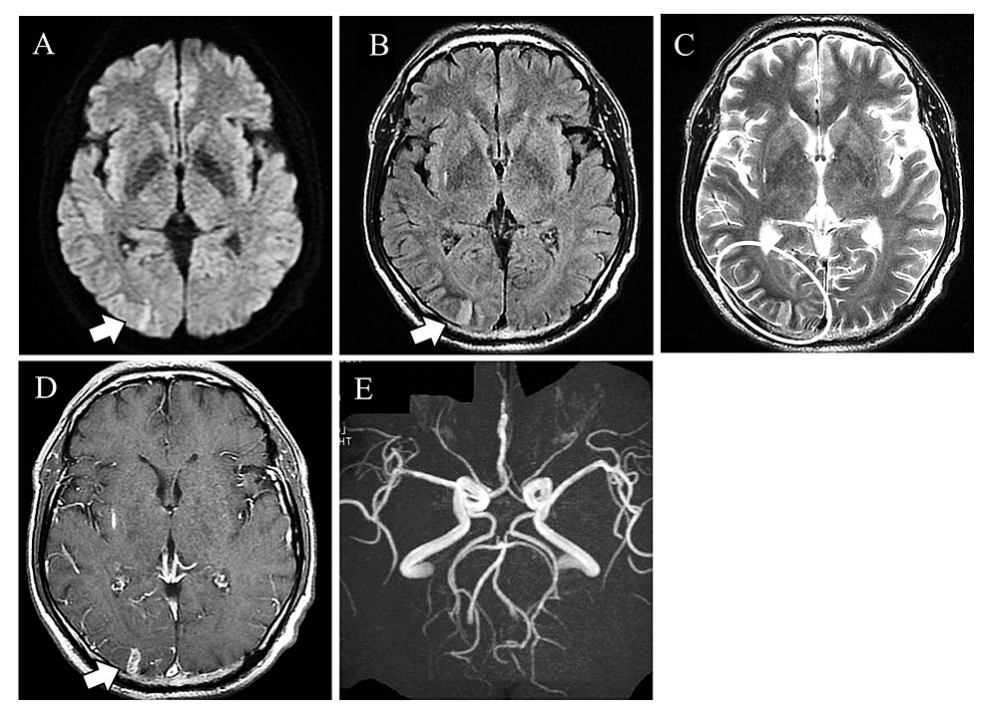
Contrast-enhanced magnetic resonance imaging (MRI). The occipital lobe showed cortical high intensity on diffusion-weighted image (A), fluid-attenuated inversion recovery (B), and a subcortical low-signal on T2 (C). The occipital lesion showed gadolinium enhancement (D). MR angiography showed no vessel stenosis or occlusion (E).

Lumbar puncture revealed elevation of the glucose level (140 mg/dL, normal, 50-75 mg/dL), but the cerebrospinal fluid pressure, protein level, and cell count were within normal range. Brain MRI revealed occipital lobe hyperintensity on the diffusion-weighted image (DWI) and fluid-attenuated inversion recovery (FLAIR) associated with subcortical T2 low signal. The occipital lobe lesion showed gadolinium enhancement. No vascular stenosis or occlusion was seen on MR angiography (Figure 1).

**Figure 2:**
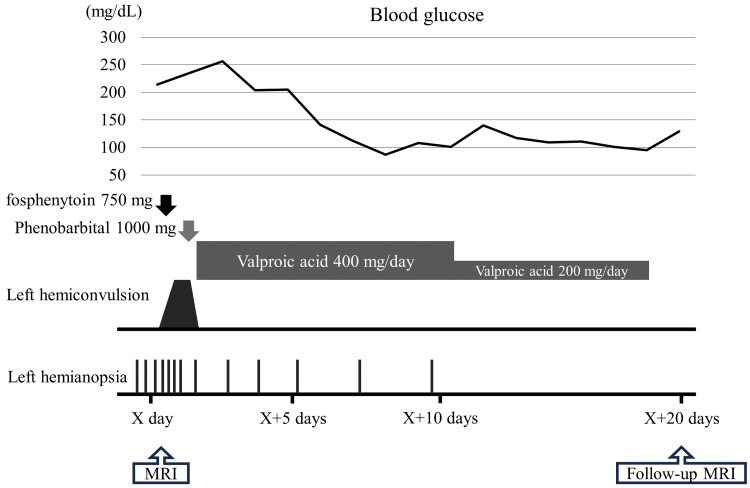
The clinical course of the patient after admission. On admission, he complained only of visual abnormalities; however, left hemiconvulsion later developed. As a result, diazepam, fosphenytoin, and phenobarbital were administered. After that, oral valproic acid was administered, and the convulsion resolved. Insulin was also administered to control blood glucose, and his visual abnormality gradually improved. X day, the date of admission; MRI, magnetic resonance imaging.

Electroencephalography was performed after anti-seizure medication administration and showed no epileptic abnormalities, but only slow waves were observed. After admission, we started insulin therapy for hyperglycemia. The patient's chief complaint at the time of admission was only visual abnormality, but after admission, seizures occurred in the left half of the body. Diazepam, fosphenytoin, and phenobarbital were administered, and the seizure ceased. Subsequently, oral valproic acid was administered, and the seizures did not recur. As his blood glucose control improved, the symptoms of visual field abnormality gradually improved and disappeared. Laboratory results showed no evidence of infection, emboli, and vascular diseases. Because the symptoms of homonymous hemianopsia and seizure improved, the anti-seizure medication was discontinued, and the patient was discharged home after 20 days of admission (Figure 2).

**Figure 3:**
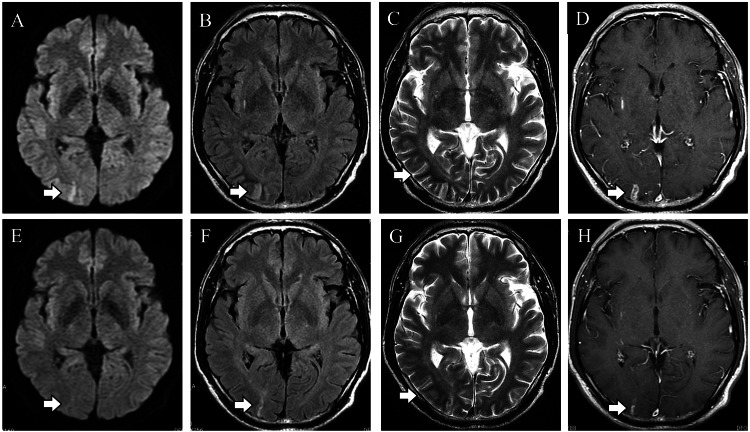
Follow-up brain magnetic resonance imaging. MRI on admission (A-D) and follow-ups (E-H). Hyperintensity lesion in diffusion-weighted imaging and fluid-attenuated inversion recovery improved. Twenty days after admission, T2 low-signal lesions disappeared, and gadolinium-enhanced lesions shrank (E-H, arrows).

Brain MRI after treatment showed improvement in high-signal lesions on DWI and FLAIR, disappearance of T2 low-signal lesions, and shrinkage of contrast-enhanced lesions (Figure 3).

## Discussion

This case presented left homonymous hemianopsia and left hemiconvulsion occurring in a severe nonketotic hyperglycemic status resulting from massive soft drink consumption. His MRI showed cortical DWI and FLAIR high signal and T2 subcortical low signal in the occipital lobe associated with gadolinium contrast enhancement. We considered cerebral infarction, PRES, epileptic change, and nonketotic hyperglycemic convulsions as possible differential diagnoses. Hyperglycemia sometimes produces occipital lobe epilepsy, which is known as NKHS [4,6,10]. In MRI studies, epileptic patients sometimes present with focal cortical edema showing DWI, T2, and FLAIR hyperintensity lesions with gadolinium contrast enhancement in the cerebral sulci [11]. Similar MRI findings are described in hyperglycemic chorea, in which the capsule and caudate nuclei show T1 high-intensity and T2 iso- to low-intensity lesions [12]. In NKHS, it has been reported that imaging typically shows reversible high signal intensity on DWI, high signal intensity on cortical T2/FLAIR, low signal intensity on subcortical T2, and leptomeningeal enhancement [13]. The MRI characteristics of this case and the differential diseases (NKHS, epilepsy, hyperglycemic chorea, PRES, and acute ischemic stroke) are shown in Table 2 [14-16].

**Table 2:** Imaging features of this case and the differential diagnoses. Imaging features are listed, referring to previous reports [14-16]. The imaging findings of this case were considered most similar to NKHS. MRI, magnetic resonance imaging; NKHS, nonketotic hyperglycemia syndrome; PRES, posterior reversible encephalopathy syndrome; FLAIR, fluid-attenuated inversion recovery; DWI, diffusion-weighted image; ADC, apparent diffusion coefficient

MRI findings	Our case	NHKS	PRES	Epilepsy	Acute ischemic stroke
Distribution of lesions	Occipital cortex and white matter	Occipital lobe predominance	Occipital lobe predominance	Cortex > white matter	Consistent with the vascular territory
T1	Iso	Iso	Low	Low	Iso-low
T2	Low	Low	High	High	High
FLAIR	Low intensity around high-intensity	Cortical high and subcortical low	High	High	High
DWI	Low	High	Iso-high	High	High
ADC	Low	Low	Low-iso-high	Low	Low
Gadolinium enhancement	Cortical/leptomeningeal enhancement	Cortical/leptomeningeal enhancement	Cortical enhancement in one-third of cases	Not common	Enhancement in the subacute period

Based on the medical history, clinical course, and laboratory findings, including contrast-enhanced MRI, this case was diagnosed as an NKHS-like condition. As for imaging findings, reversible DWI high signal, cortical T2/FLAIR high signal, subcortical T2 low signal, and leptomeningeal enhancement have been reported in NKHS [8,17]. The patient was subsequently confirmed as NHKS by the resolution of symptoms and imaging abnormalities with glucose management.

Hyperglycemia possibly induces convulsions due to cytotoxicity caused by increased cellular osmolarity and the disruption of the citric acid cycle. It is known that hyperglycemia predisposes to convulsions. Huang et al. reported that high HbA1c is associated with increased frequency and severity of seizures [18]. However, among hyperglycemia, convulsive seizures are more common in nonketotic hyperglycemia [19] because ketosis is protective against convulsions [20]. Also, there are other nonketotic involuntary movements, such as hyperglycemia-induced involuntary movement. This manifestation includes tremors, opsoclonus, hemifacial spasm, parkinsonism, myoclonus, dystonia, torticollis, and restless leg syndrome [15]. Based on this information, we diagnosed this case as having NKHS triggered by marked hyperglycemia, resulting in homonymous hemianopsia and convulsions. It should be noted that NKHS is reported to be characterized by a predominance of occipital lobe involvement [4,10], which results in characteristic syndromes such as the intermittent homonymous hemianopsia observed in our case.

## Conclusions

Here, we report our characteristic case with a case of epileptic homonymous hemianopsia associated with severe hyperglycemia. Brain MRI of our case showed characteristic subcortical T2 hypointensity lesions and gadolinium contrast enhancement in the brain parenchyma. Those findings were consistent with NKHS. It should be noted that NKHS can produce occipital lobe epilepsy, which can result in visual symptoms. We reported this case because we considered it necessary to accumulate similar cases in the future. Also, we have compiled a table of diseases that presents a similar picture. We trust that this table will be of use to clinicians.
